# Identification of Influenza A/PR/8/34 Donor Viruses Imparting High Hemagglutinin Yields to Candidate Vaccine Viruses in Eggs

**DOI:** 10.1371/journal.pone.0128982

**Published:** 2015-06-11

**Authors:** Adam Johnson, Li-Mei Chen, Emily Winne, Wanda Santana, Maureen G. Metcalfe, Guaniri Mateu-Petit, Callie Ridenour, M. Jaber Hossain, Julie Villanueva, Sherif R. Zaki, Tracie L. Williams, Nancy J. Cox, John R. Barr, Ruben O. Donis

**Affiliations:** 1 Influenza Division, National Center for Immunization and Respiratory Diseases, Centers for Disease Control and Prevention, Atlanta, Georgia, United States of America; 2 Division of Laboratory Sciences, National Center for Environmental Health, Centers for Disease Control and Prevention, Atlanta, Georgia, United States of America; 3 National Center for Emerging and Zoonotic Infectious Diseases, Centers for Disease Control and Prevention, Atlanta, Georgia, United States of America; University of Georgia, UNITED STATES

## Abstract

One of the important lessons learned from the 2009 H1N1 pandemic is that a high yield influenza vaccine virus is essential for efficient and timely production of pandemic vaccines in eggs. The current seasonal and pre-pandemic vaccine viruses are generated either by classical reassortment or reverse genetics. Both approaches utilize a high growth virus, generally A/Puerto Rico/8/1934 (PR8), as the donor of all or most of the internal genes, and the wild type virus recommended for inclusion in the vaccine to contribute the hemagglutinin (HA) and neuraminidase (NA) genes encoding the surface glycoproteins. As a result of extensive adaptation through sequential egg passaging, PR8 viruses with different gene sequences and high growth properties have been selected at different laboratories in past decades. The effect of these related but distinct internal PR8 genes on the growth of vaccine viruses in eggs has not been examined previously. Here, we use reverse genetics to analyze systematically the growth and HA antigen yield of reassortant viruses with 3 different PR8 backbones. A panel of 9 different HA/NA gene pairs in combination with each of the 3 different lineages of PR8 internal genes (27 reassortant viruses) was generated to evaluate their performance. Virus and HA yield assays showed that the PR8 internal genes influence HA yields in most subtypes. Although no single PR8 internal gene set outperformed the others in all candidate vaccine viruses, a combination of specific PR8 backbone with individual HA/NA pairs demonstrated improved HA yield and consequently the speed of vaccine production. These findings may be important both for production of seasonal vaccines and for a rapid global vaccine response during a pandemic.

## Introduction

Vaccination is the major medical intervention to mitigate the public health impact of illness due to influenza. The majority of the seasonal influenza vaccines produced globally are trivalent inactivated products that are manufactured in large scale by propagating selected high-growth reassortant (HGR) viruses in embryonated chicken eggs. Virions are subsequently chemically inactivated, purified and generally split into subviral antigens which are formulated with or without adjuvants and dispensed into multi-dose vials or individual syringes [1]. The complexity of the fertile egg supply chain and the specialized manufacturing facility requirements limit options to expand production in response to a pandemic, underscoring the critical importance of maximizing the productivity of HGRs used in the current manufacturing processes. Because the success of vaccination depends on immunizing the population before the new virus spreads widely [2,3,4], rapid pandemic vaccine development, manufacturing and delivery are essential.

The qualitative and quantitative growth characteristics of the HGR vaccine virus seeds used in manufacturing are widely recognized as major determinants of the number of vaccine doses obtained from each egg [5]. Most wild-type seasonal influenza viruses encoding the recommended HA and NA antigens for immunization grow poorly in eggs even after sequential passages in the laboratory [6]. In the late 1960's, Kilbourne *et al*. [7] mitigated this problem by exploiting the exceptional replication efficiency of the laboratory-adapted A/Puerto Rico/8/1934 (PR8) virus in eggs through genetic reassortment [6]. This technique is used to produce viruses containing the surface genes (HA and NA) from the epidemic/pandemic virus of interest and most of the "internal genes" (PB1, PB2, PA, NP, M, NS) from PR8; such reassortants generally have virus yields exceeding those of the parental wild-type virus donor. These candidate vaccine viruses (CVV) are designated as high growth reassortant (HGR) or, when appropriate, 6:2 genotype reassortant (6:2R) viruses. Although efficient replication of HGR viruses in eggs is a multi-genic trait involving surface (HA and NA) as well as internal genes, the M gene of the PR8 donor is invariably required [8].

Most of the inactivated seasonal influenza vaccines manufactured since the early 1980's utilize PR8-derived HGR viruses [5], generally with 6:2 genotypes, for production of the type A components. Several laboratories developing HGR viruses for vaccine production have used different lineages of PR8 viruses, with distinct nucleotide and amino acid polymorphisms arising as a consequence of diverse passage histories [9] which may impact the antigen yields of HGR viruses in manufacturing. Furthermore, HA antigen yields from 6:2R viruses with HA and NA genes from influenza viruses of avian or swine origins have been highly variable and frequently fall below the acceptable range for manufacturing vaccine in eggs, which is generally no less than 10 mg of total viral proteins from the allantoic fluid of 100 eggs [10,11]. Complex influenza virus phenotypes, such as replication in chicken embryos, are invariably determined by multiple genetic loci that encode critical functions such as virus attachment to receptors and entry into host cells (encoded mainly by the HA and NA genes) as well as gene expression and genome replication (encoded by the 6 internal genes) [12,13,14,15,16,17]. Functional compatibility between HA and NA is critical to achieve efficient replication in the target host. Likewise, compatibility among replicase/transcriptase genes and accessory genes (M and NS) is essential for efficient virus growth. Although these two functional units operate somewhat autonomously, there is evidence of genetic interactions between these two broadly defined functions [12,13,14,15,16,17]. Conducting a detailed analysis of the functional properties of available PR8 internal gene donors and the most relevant HA-NA gene subtype/lineage combinations is necessary to determine whether certain gene combinations can impart high growth in eggs.

In this study, we selected three different PR8 virus lineages which have been used as the donors of internal genes for development of reassortant seasonal and pre-pandemic candidate vaccine viruses by WHO Influenza Collaborating Centers or Essential Regulatory Laboratories [9,14,18,19,20]. We evaluated the growth (yield) characteristics of different lineages of PR8 donor viruses when combined with surface genes from a wide range of potentially pandemic viruses to inform rapid development of HGR viruses with the highest possible antigen yields in eggs. To this end, we analyzed the viral antigen yield of three lineages of PR8 donor viruses with HA and NA genes from nine viruses that represent divergent North American and Eurasian lineages of viruses from avian, swine and human hosts. Our findings have important implications for strengthening vaccine preparedness to achieve effective pandemic mitigation.

## Materials and Methods

### Origin of influenza virus genes used to generate reassortant viruses

Influenza A viruses were used as donors of HA, NA and internal genes (Tables 1 and 2).

**Table 1:** Amino acid variation among viral proteins encoded by the PR8 internal genes.^†^

Gene	Amino acid	Backbone-A	Backbone-B	Backbone-C
PB2	105	M	I	I
	251	K	R	R
	299	K	R	R
	360	S	Y	Y
	504	V	I	I
	702	R	K	K
PB1	175	K	N	N
	205	I	M	M
	208	R	K	K
	216	G	S	S
	563	R	I	R
PB1-F2	59	K	R	R
	60	Q	R	R
PA	158	R	K	K
	550	L	I	I
PA-x	158	R	K	K
	212	A	V	A
NP	116	I	I	M
	353	V	L	L
	425	V	I	I
	430	T	N	N
M2	27	A	T	T
	39	I	T	T
NS1	23	V	A	A
	55	E	K	E
	101	E	D	D
NS2	26	E	E	G
	89	V	I	I

†References: [9,14,19]

**Table 2:** HA and NA genes utilized in reassortment with selected PR8 donors along with IDMS peptides used in this study.^†^

Virus name	Subtype	Lineage	Host Adaptation	Peptides used for HA Quantification by IDMS	Peptides used for NA Quantification by IDMS
**A/Puerto Rico/8/1934**	H1N1	Donor A, B and C	ferret, chicken embryo	EQLSSVSSFER TLDFHDSNVKVNSVIEK	YNGIITETIK GDVFVIR
**A/New York/18/2009**	H1N1 pdm	Global	swine and human	EQLSSVSSFER TLDYHDSNVKVNSVIEK	YNGIITDTIK GDVFVIR
**A/swine/Missouri/ 2124514/2006**	H2N3	North American	swine and avian	VNSVIEKTLDFHDSNVK EFNNLER	SGFEIIK
**A/Hawaii/7/2009**	H3N2	Global	human seasonal	STQAAIDQINGKDEALNNR EFSEVEGR	SGYSGIFSVEGK
**A/Bangladesh/5071/2011**	H3N2	Global	human seasonal	STQAAIDQINGKDEALNNR EFSEVEGR	SGYSGIFSVEGK
**A/Indiana/10/2011**	H3N2v	North American	swine	STQAAINQITGKNEALNNR EFSEVEGR	SGYSGIFSVEGK
**A/Vietnam/1203/2004**	H5N1	Eurasian,clade 1	avian	TLDFHDSNVKEEISGVK LVLATGLREFNNLER	YNGIITDTIK GDVFVIR
**A/Hubei/1/2010**	H5N1	Eurasian,clade 2.3.2.1	avian	EEISGVKLVLATGLR EFNNLER	YNGIITDTIK GDVFVIR
**A/New York/107/2003**	H7N2	North American	avian	VNTLTERSTQSAIDQITGK IQIDPVK	SGYETFR
**A/Shanghai/2/2013**	H7N9	Eurasian	avian	VNTLTERFVNEEALRSTQSAIDQITGKIQIDPVK	IGESSDVLVTRFYALSQGTTIRVPNALTDDR

†Peptides used for NP quantitation by IDMS were LIQNSLTIER and GVFESLDEK, whereas M1 quantitation used EITFHGAK.

### Generation and sequence analysis of reassortant viruses by reverse genetics

Reassortant viruses were generated from plasmids by a reverse genetics approach. The viral cDNAs were cloned into a plasmid vector under control of the human polymerase I promoter and the mouse RNA polymerase I terminator (herein pPolI vector). Reverse genetics plasmids encoding HA and NA surface genes and 6 internal genes were transfected into HK 293T cells using Lipofectamine 2000 (Life Tech). For hemagglutinin genes derived from H5N1 highly pathogenic virus, including A/Vietnam/1203/2004 and A/Hubei/1/2010, overlapping PCR mutagenesis was performed to remove the polybasic amino acid. Viruses derived by plasmid transfection of HK293 cells were propagated in 10–11 day old embryonated eggs and incubated at 35–37°C for 48 hrs. H1N1pdm, H5N1, and H7N9 CVV with PR8-A were generated for pandemic preparedness purposes[56].

Mutations in the HA genes were introduced by site directed mutagenesis (QuikChange Multi Site-directed Mutagenesis Kit: Agilent Technologies; Santa Clara, CA) and confirmed by sequence analysis.

Total RNA was extracted from allantoic fluid using the QiAmp Viral RNA minikit (Qiagen; Valencia, CA) and reverse transcribed to cDNA and amplified using a one-step reaction system (One step RT-PCR kit: Qiagen) and sequence specific primers. Sequence analysis of the resulting DNA amplicons served as templates for automated sequencing on an Applied Biosystems 3130 genetic analyzer, using cycle sequencing dye terminator chemistry (BigDye terminator v3.1 cycle sequencing kit; Life Tech).

### Morphologic analysis by EM

Reassortant viruses in allantoic fluid were fixed in 2.5% paraformaldehyde and prepared for NSEM. For each virus sample, 2 μl was pipetted onto a 1% alcian blue treated formvar-carbon coated 300 mesh nickel grid (Electron Microscopy Sciences). Samples were incubated overnight on grids in a refrigerator at 4°C. Following overnight incubation, samples were blotted, rinsed with 1% bacitracin (Gregory & Pirie, 1973), blotted and stained with 5% ammonium molybdate [pH 6.9] with 1% trehalose (w/v) (Harris et al., 2006). The stain was immediately blotted after applying and grid was then allowed to air dry prior to viewing at the transmission electron microscope (120 kV, BioTwin, FEI, Hillsboro, OR)[57,58]. For each 6:2R sample, the maximum and minimum diameters (nm) of 250 influenza virions were measured using AMT software (Advanced Microscopy Techniques, Corp.). Measurements were imported into Microsoft Excel to calculate the number of spherical and non-spherical virions based on the measured maximum diameter to minimum diameter ratio.

### Virus propagation in eggs and concentration

All of the reassortant viruses were grown in 10–11 day-old embryonated hens’ eggs at 35–37°C for 48 hrs (~62 hrs for H7N9 virus). Allantoic fluid was harvested from the chilled eggs and clarified at 15,180 g for 10 min at 4°C (Sorvall SLA-1500 rotor). The supernatant was collected and inactivated with ß-propiolactone (BPL) for approximately 24 hrs at 4°C before further purification. The inactivated virus was pelleted by centrifugation at 38,890 g for 3 hrs at 4°C (Sorvall A621 rotor). Virus pellets were resuspended overnight in phosphate buffered saline pH 7.40 (PBS) and loaded onto a 30%/55% sucrose gradient. The gradient was centrifuged at 90,235 g (Sorvall AH629 rotor) for 14hrs. The virus fractions were harvested and further pelleted at 131,101 g for 2.5 hrs. Each virus was propagated and purified at least twice independently using a different batch of eggs. The three viruses sharing HA and NA surface genes were always analyzed as a set.

### Quantification of viral proteins in concentrated viruses by BCA

A microplate Pierce BCA assay kit was used to measure the total protein content of the purified virus concentrate. For each sample, NP40 was added to a final concentration of 0.25%. Bovine serum albumin (BSA) provided in the kit was used for the standard curve, and absorbance was read at 562 nm. The BCA assay was performed according to manufacturer’s instructions.

### Quantification of HA by SDS-PAGE and densitometry analyses

Approximately 10 μg of each virus concentrate was deglycosylated with PNGaseF (New England Biolabs) according to the manufacturer’s instructions. The deglycosylated samples were loaded on a 10% Bis-Tris precast NuPAGE polyacrylamide gel (Life Tech) and electrophoresed in NuPAGE MOPS SDS running buffer under reducing conditions. Two-fold serially diluted BSA was prepared and loaded on the same gel as the standards for quantitation of virus protein bands. The protein gels were stained with colloidal Coomassie blue (Life Tech), imaged with an ImageScanner III, and quantified using ImageQuant software (GE Healthcare). The HA protein content of each deglycosylated virus sample was calculated by comparison to the BSA standards with known quantities on the same gel.

### Quantification of HA, NP, and M1 by IDMS

Isotope dilution mass spectrometry (IDMS) was performed as described previously for quantification of HA and NA proteins in seasonal H3N2, H1N1pdm09 [59], avian H5N1 [60], H7N9, H7N2, and H7N7 [61] strains in purified virus samples. These protocols were modified to quantify the NP and M1 proteins of influenza A and influenza B viruses (manuscript in preparation). Briefly, the IDMS method involves enzymatic digestion of viral proteins and the specific detection of evolutionarily conserved target peptides. Once the target peptides are identified, a labeled analog of the peptide is synthesized by incorporating an amino acid proximate to the C-terminus with ^13^C and ^15^N to generate a peptide that is heavier in mass than the native target peptide as described previously [60]. The target peptides used for HA, NA, NP, and M1 are shown in Table 2. Tryptic digestion of the viral proteins was followed by separation of the peptides on an Agilent 1200 Capillary LC system (Agilent, Santa Clara, CA) and analyzed on a Thermo TSQ Vantage triple quadrupole mass spectrometer (Thermo Scientific, Waltham, MA).

### Statistical analyses

Data were analyzed by 1-way ANOVA with Fisher’s LSD post-hoc analysis using the JMP software package (Version 10, SAS Inc.).

## Results

### Genetic analysis of three lineages of PR8 donor viruses

Three lineages of PR8 influenza viruses (PR8-A, PR8-B and PR8-C) used by global public health laboratories to produce HGR viruses for vaccine manufacturing were selected for this study (Table 1) [9,14,15,20,21,22]. The proteins encoded by the internal genes (PB2, PB1, PB1-F2, PA, PA-X, NP, M1, M2, NS1 and NS2) revealed multiple amino acid differences between PR8-A and PR-B or PR8-C genes, whereas PR8-B and PR8-C were more similar to each other (Table 1). The ten proteins encoded by the six internal genes of PR8-A differed from those of PR8-B and PR8-C at 26 and 25 positions, respectively, but only 5 amino acid differences were noted between PR8-B and PR8-C (Table 1).

### Virologic and morphologic analysis of 6:2 reassortant viruses

To evaluate the antigen yields of HGR viruses with the different PR8 lineages bearing a broad range of HA and NA genes, multiple subtypes of Eurasian or North American lineages were selected, including viruses of avian, swine and human origin (Table 2). PR8 6:2R viruses were generated from reverse genetics plasmid sets comprising the wild-type HA and NA genes from avian viruses or human seasonal viruses. Utilizing nine selected HA-NA gene pairs and three lineages of PR8 donor viruses, a total of 27 reassortant viruses were generated by reverse genetics, all with 6:2 genotypes. Efficient growth in eggs required HA mutations in several strains: A/New York /18/2009 D222G, A/Hawaii/07/2009 H183L, A/Bangladesh/5071/2011 H156Q/G186V/S219Y, and A/Indiana/10/2011 L194I, based on the sequences of parental viruses isolated in eggs. Full genome sequences of the second egg passage of these 6:2R viruses recovered from transfected cells indicated absence of coding changes in these viral genomes as compared to the sequences of the cDNA cloned in plasmids.

The replicative properties of reassortant viruses were analyzed using the second egg passage of plasmid DNA-transfected cell supernatants. All reassortant viruses replicated efficiently in eggs, reaching infectious titers between 1.0 x 10^8^ and 2.4 x 10^10^ EID_50_/ml (S1 Fig). Hemagglutinating unit (HAU) titers ranged from 128 to 4096, in overall agreement with the infectivity results (S1 Fig).

**Fig 1:**
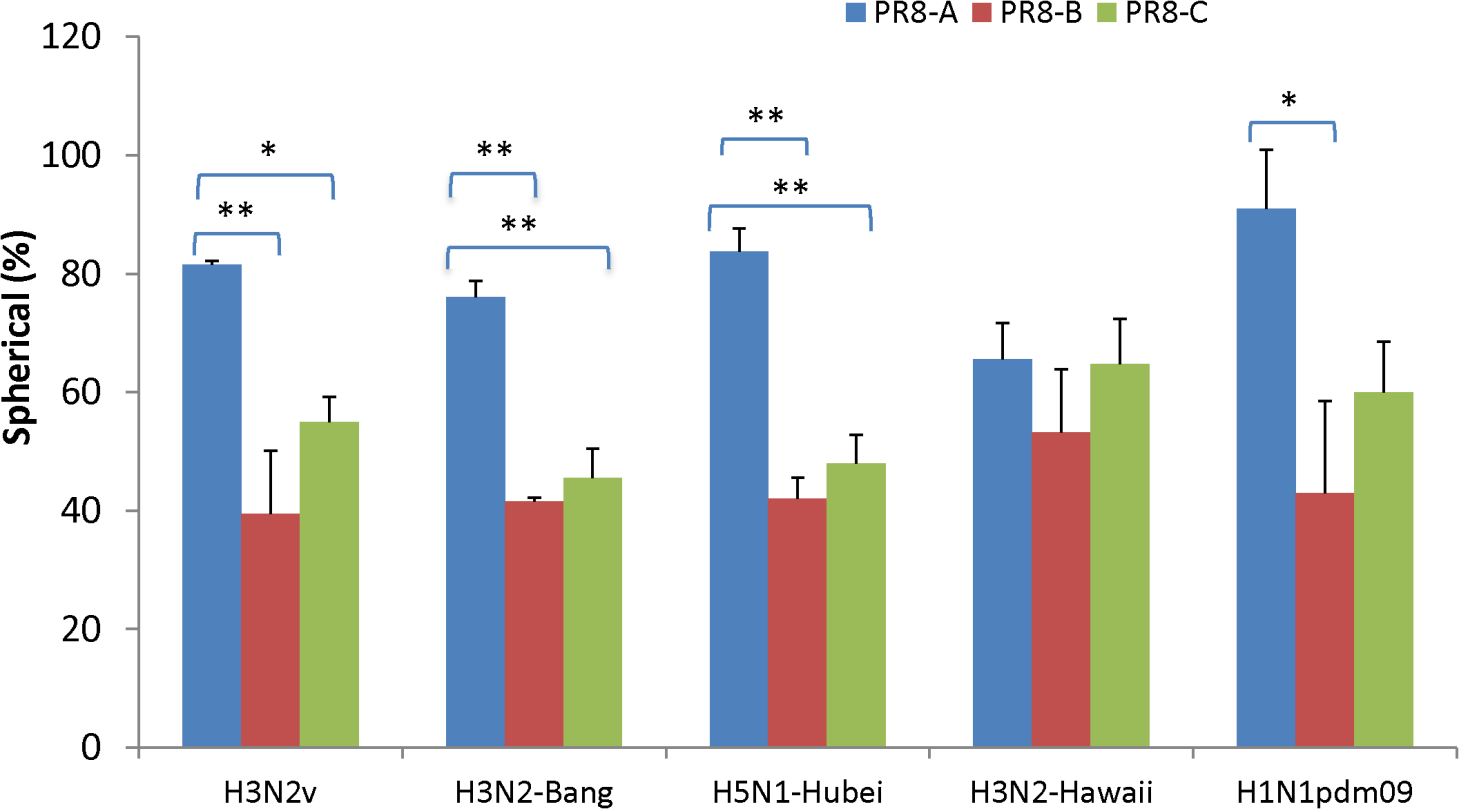
Percentage of spherical virions from reassortant viruses. Reassortant viruses were propagated in embryonated eggs, fixed in 2.5% paraformaldehyde and analyzed by NSEM. A total of 250 virus particles from each reassortant virus were analyzed. Virus particle with axial ratio <1.5 is scored as spherical virion. Statistical significance was assessed as described in Materials and Methods (*, *P* < 0.05; **, *P* < 0.01).

Previous studies suggested that incompatibility between PR8 and very divergent HA-NA genes may result in release of pleomorphic or filamentous virions [23,24,25,26,27] with unpredictable impact on manufacturing steps such as filtration or centrifugation. To investigate the possible impact of PR8 internal genes on virion morphology, allantoic fluid harvests from a subset of five representative 6:2R viruses were analyzed by negative-stain electron microscopy (NSEM) (Fig 1 and S2 Fig). The approximate percentage of spherical virions (defined as virions whose ratio of maximum diameter (nm) to minimum diameter (nm) is between 1 and 1.5) ranged from 41.5% to 91% on average for the 15 viruses analyzed (5 sets of surface genes with 3 different PR8 internal genes) (Fig 1). With the exception of A/Hawaii/7/2009 (H3N2) 6:2R, viruses with PR8-A internal genes yielded a consistently higher percentage of spherical particles (overall mean 79.6%) as compared to PR8-B and PR8-C (43.8% and 54.6%, respectively) (P<0.05) (Fig 1).

### Viral protein yield of 6:2 reassortant viruses with selected PR8 internal genes

**Fig 2:**
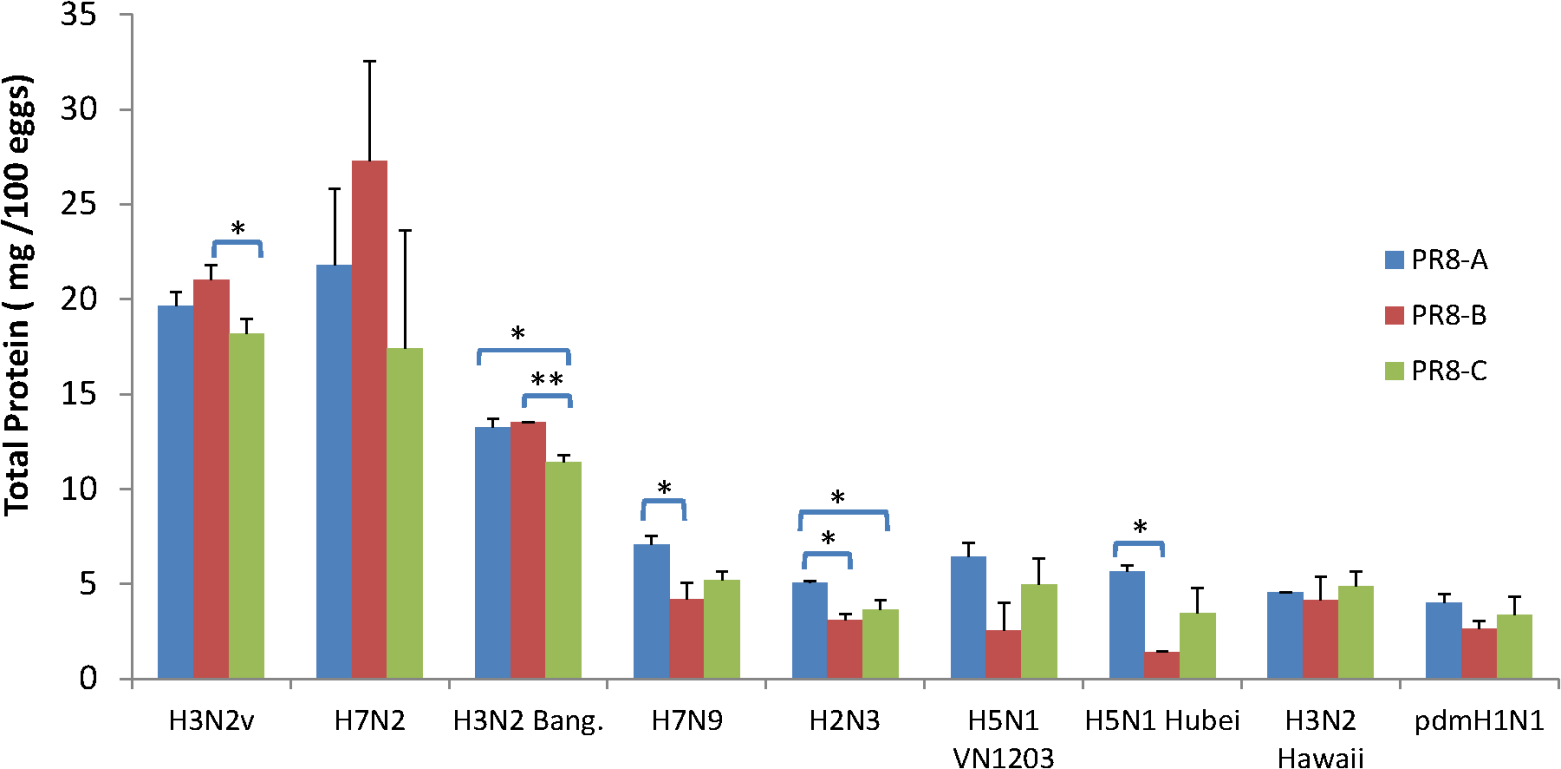
Total viral protein yields in virions concentrated from allantoic fluid. Reassortant viruses were propagated in embryonated eggs and purified from allantoic fluid by sucrose density gradient ultracentrifugation. Each virus was propagated and purified at least twice independently using a different batch of eggs. Total viral protein in purified virus samples was determined by BCA as described in Materials and Methods. Statistical significance: *, *P* < 0.05; **, *P* < 0.01.

Although infectivity and HA assay titers in allantoic fluids are informative to estimate 6:2R virus performance in vaccine manufacturing, they are not definitive predictors of viral antigen yield after chemical inactivation and purification methods used in production (e.g. ultracentrifugation and/or chromatography). To estimate the potential vaccine antigen yields under manufacturing conditions, we evaluated HA content in inactivated virions concentrated from allantoic fluid by differential and equilibrium ultracentrifugation in sucrose gradients. Previous reports showed that the minimum total viral protein requirement for manufacturing is approximately 10 mg/100 eggs [15]. In this study, the total virion protein yields from 6:2R viruses determined by BCA ranged from a high of 27 mg/100 eggs to a low of 1.4 mg/100 eggs (Fig 2). The A(H3N2)v, A(H7N2), and A/Bangladesh/5071/2011 (H3N2) viruses formed a cluster with yields of >11 mg/100 eggs, exceeding the minimum yield required for manufacturing and therefore were designated high antigen yield (HAY) viruses. In contrast, the remaining 6 viruses were characterized by total protein yields ≤6.5 mg/100 eggs and were designated low antigen yield viruses (LAY). The three HAY 6:2R viruses revealed that PR8-B derived viruses yielded higher total protein than PR8-C viruses in 2/3 of instances (P<0.05) (Fig 2). In contrast, comparisons within LAY 6:2R virus sets revealed that yields from PR8-A viruses were often higher than those of PR8-B viruses (3/6 6:2R sets with P<0.05) (Fig 2).

**Fig 3:**
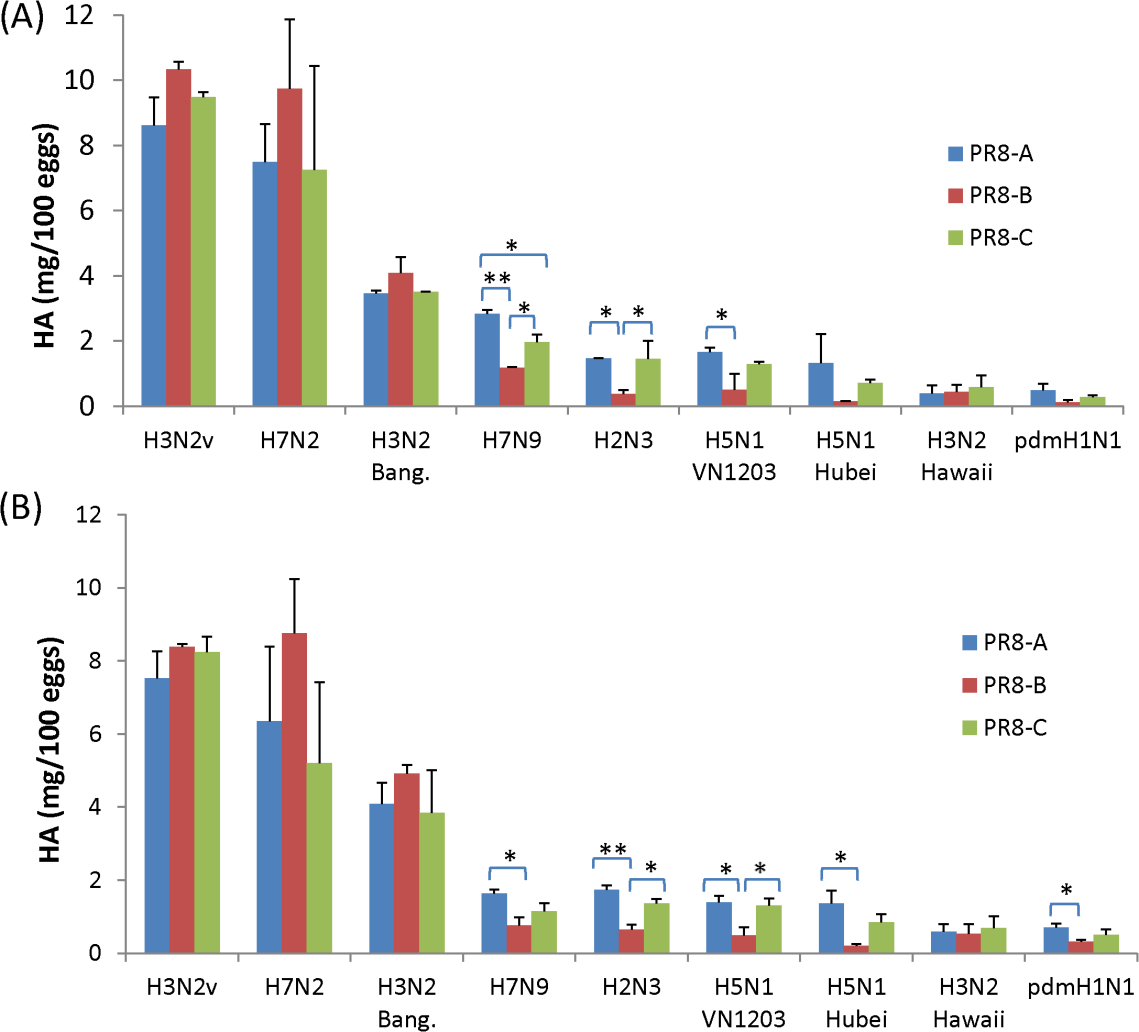
Total HA yield in virus concentrates determined by IDMS and SDS- PAGE/densitometer analysis. Reassortant viruses were propagated in embryonated eggs and purified from allantoic fluid by sucrose density gradient ultracentrifugation. Each virus was propagated and purified at least twice independently using a different batch of eggs. Total HA in concentrated virus samples was determined by IDMS (A) and SDS-PAGE/densitometry (B) as described in Materials and Methods. Statistical significance: *, *P* < 0.05; **, *P* < 0.01.

To further quantify the antigen yield of the 6:2R viruses, we analyzed the purified viruses by isotope dilution mass spectrometry (IDMS) and SDS-PAGE/densitometry analysis. IDMS quantification revealed clustering of viruses into HAY (>3.4 mg/100 eggs; H3N2 Bang.) and LAY (<2.8 mg/100 eggs; H7N9) groups with almost the same distribution as determined by total virion protein assays (Fig 3A). Similarly, the total HA content of 6:2R virions concentrates determined by SDS-PAGE/densitometry (Fig 3B) ranged from <1.6 mg/100 eggs (H7N9) for LAY viruses to >3.8 mg/100 eggs (H3N2 Bang.) among HAY viruses. The HA yields determined by SDS-PAGE assays were slightly lower than those obtained by IDMS, possibly due to the lower basic amino acid content of influenza HA protein relative to BSA, resulting in decreased Coomassie blue stain uptake. However, the relative HA yields of viruses with different PR8 backbones remained consistent across assays. Comparisons within LAY 6:2R sets revealed that yields from PR8-A viruses were often higher than those of PR8-B viruses (3/6 6:2R sets with P<0.05) and in some cases yields from PR8-C viruses also were higher than those of PR8-B (P<0.05).

### Relative HA content of concentrated virus samples

**Fig 4:**
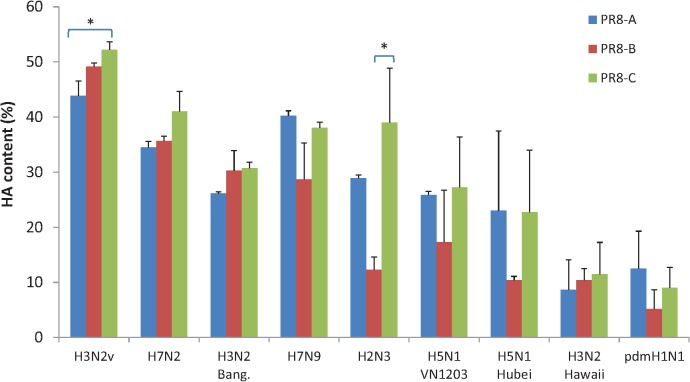
Relative HA content of concentrated virus samples. The relative HA content of each reassortant virus was calculated based on the ratio of the HA (determined by IDMS) to the total protein (determined by BCA). Statistical significance: (*, *P* < 0.05; **, *P* < 0.01).

**Fig 5:**
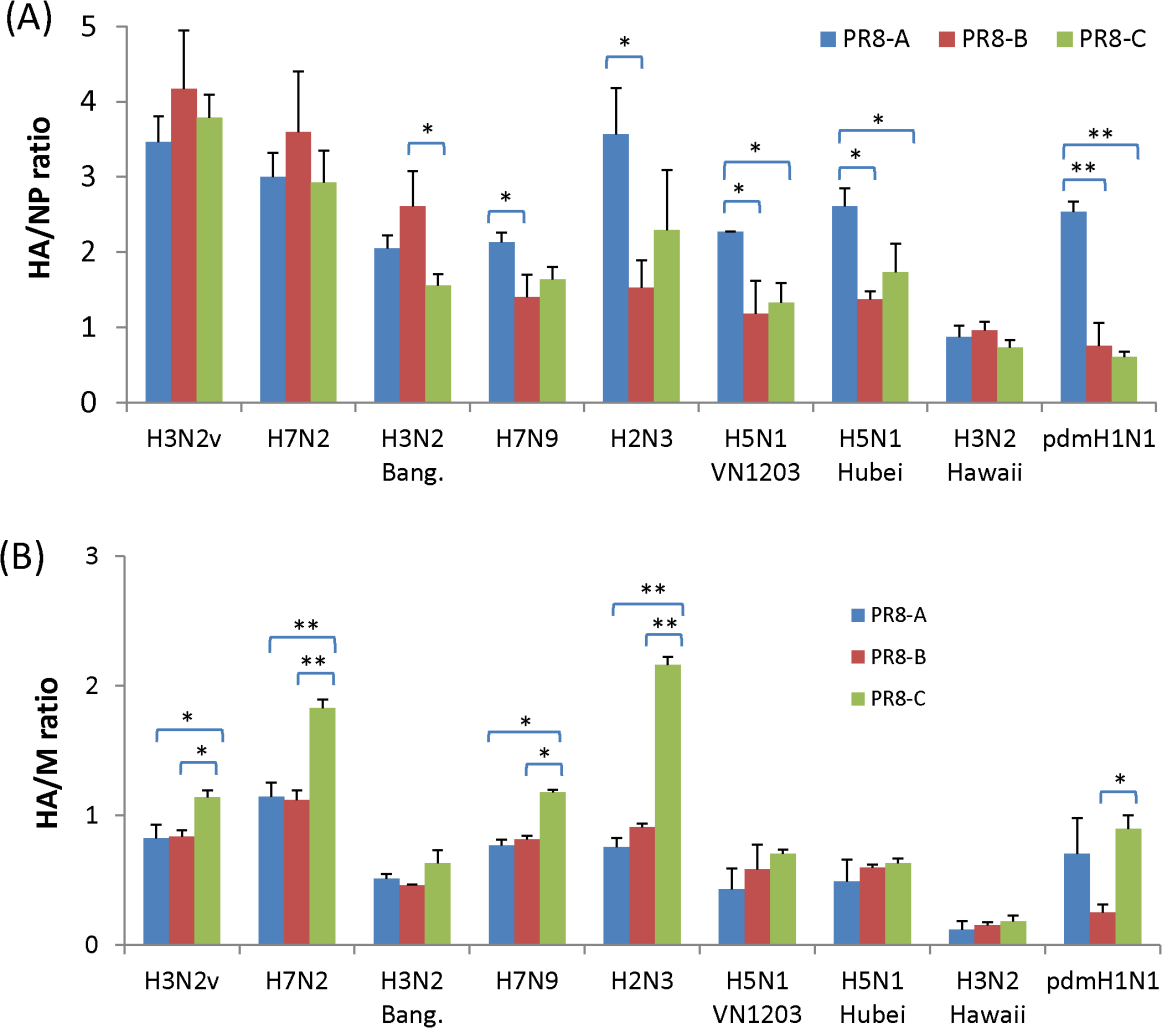
Ratio of viral protein from virions concentrated from allantoic fluid. The molar ratio of (A) HA/NP and (B) HA/M1 of the concentrated viruses. The HA, NP and M yield of the viruses were determined by IDMS. Statistical significance: (*, *P* < 0.05; **, *P* < 0.01).

Previous reports demonstrated that the low antigen yield from A/Vietnam/1194/04 (H5N1) NIBRG-14 reassortant virus in eggs was caused by inefficient incorporation of HA into virions [13,28,29]. To address this question, we calculated the percentage of HA determined by IDMS relative to total viral protein determined by BCA (Fig 4). The mean HA content of purified virus samples ranged from 8.5% to 48.4%. The impact of PR8 on relative HA content was not statistically significant (Fig 4). The potential differences in HA incorporation into virions were obscured by the combined variance of BCA and IDMS determinations. To investigate whether virion HA content varied in relation to other proteins, we quantified amount of NP and M protein in concentrated viruses by IDMS using a custom set of synthetic isotopically labeled peptides (Table 2) as internal standards, and calculated their HA/NP and HA/M1 ratios. The highest HA/NP ratios for the LAY viruses were noted among PR8-A internal genes with H7N9, H2N3, H5N1 and H1N1pdm09 surface genes (Fig 5A). Interestingly, a higher HA/M1 ratio is observed for the viruses with the internal genes derived from PR8-C (Fig 5B; H3N2v, H7N2, H7N9, H2N3, and pdmH1N1). The increased ratio of HA/NP or HA/M1 for PR8-A and PR8-C-derived viruses coincided with improved HA content and HA antigen yield, suggesting a possible increased incorporation of HA antigen into virions.

## Discussion

Candidate reassortant viruses developed to manufacture licensed vaccines for pandemic preparedness or response are designed to meet two critical requirements: high antigen yield in eggs and adequate biosafety. The latter is ascertained by reduction in virulence and/or transmissibility in animal models [30,31]. Reassortment between influenza A viruses with pandemic potential and a PR8 donor virus has been adopted without exception because it imparts both virulence attenuation and the highest known replication ability in embryonated eggs. Although pathogenicity studies in mice and ferret models have established the safety of 6:2R viruses evaluated to date [32], suboptimal antigen yield in eggs is not uncommon. The low antigen yields of the A/California/7/2009 A(H1N1)pdm09 vaccine viruses caused delays in rapidly manufacturing large quantities of vaccine, and thus in achieving effective vaccine coverage in high-risk groups [10] and illustrated the magnitude of the challenge. The impact of low vaccine antigen yields on the effectiveness of pandemic mitigation through vaccination [2,33] provided a strong motivation for studies aimed at improving the generation of 6:2R viruses for rapid and efficient vaccine manufacturing in eggs [34].

Understanding the performance of three different PR8 virus lineages upon reassortment with nine HA-NA gene pairs representing diverse subtypes and lineages of high public health significance would establish a baseline to inform future candidate vaccine development for pandemic responses. The resulting panel of 27 viruses provided an opportunity to investigate associations between surface and internal genes, and their antigen yields based on parameters such as total viral protein or amount of HA antigen produced in eggs. A striking linkage between HA antigen yield and the source of HA-NA surface protein-coding genes was observed regardless of the PR8 gene source, reaching up to 65-fold differences when comparing 0.15 mg HA/100 eggs (by IDMS) from H5N1-Hubei to 9.5 mg HA/100 eggs from H7N2-NY, confirming previous reports [12,13,14,15,16,17,35]. The variability among HA antigen yields re-emphasized that virus surface genes are key determinants of viral growth in embryonated eggs, in agreement with other lines of evidence, such as positive selection in the HA and NA of candidate vaccine viruses selected for increased yields in eggs [36,37,38,39]. In addition, closely related HA-NA surface genes such as the two A(H3N2) and A(H5N1) viruses can impart substantially different yields. Therefore, when available, parallel evaluation of several related HA and NA genes from co-circulating viruses with equivalent antigenicity using rapid rescue techniques might provide significant gains in antigen yield and accelerate the production of vaccines to mitigate an emerging pandemic[40].

Previous studies have demonstrated the importance of PR8 internal genes for optimal replication of 6:2R viruses in eggs [9,28,41]. The association between the presence of specific PR8 internal genes and HA yields was most pronounced among the LAY 6:2R viruses, reaching 5- to 7-fold differences in hemagglutinin yield by IDMS assays for H5N1-Hubei with internal genes from PR8-A or C versus PR8-B (Fig 3A). Notably, the PR8 virus donor associated with lowest antigen yields in LAY 6:2Rs (i.e. PR8-B in H5N1 or H2N3 reassortants) mediated the best performance among the HAY 6:2R viruses, such as H7N2, H3N2v and H3N2 viruses, suggesting that functional interactions between HA-NA and PR8 genes are mediated by mutually exclusive elements in the viruses analyzed. This finding is consistent with several earlier studies concluding that functional compatibility between HA-NA (so-called “surface genes”) and PR8 internal genes is an important determinant of antigen yield from reassortant viruses in eggs [12,13,14,15,16,17,42,43]. Taken together, these results indicated that PR8 lineages A (or C) impart the highest hemagglutinin yield for 6:2R virus with low initial antigen yields in eggs. Alternatively, the PR8-B might provide superior hemagglutinin yield for other 6:2R viruses with high growth characteristics.

Previous studies established that the final HA yield from a reassortant virus used in vaccine manufacturing results not only from the quantity but also from the quality of virions produced [1]. The efficiency and kinetics of virion assembly and release into the allantoic fluid is determined by cell entry, replication and budding leading to efficient spread within the allantoic sac. The mean percentage HA determined by IDMS relative to total protein (BCA) in 6:2R virus samples revealed smaller differences among the HAY viruses (26% to 52%) than among the LAY counterparts (5% to 40%) (Fig 4). The 20–26% HA determined for A/Vietnam/1203/2004 6:2R in this study is comparable to that reported for the almost identical reassortant A/Vietnam/1194/2004 [44]. In contrast, the low HA content (~5–12%) of the A(H1N1)pdm09 and A/Hawaii (H3N2) 6:2R viruses might seem to compromise viral infectivity but previous reports suggested that virions with low HA content could support virus fusion and entry [45,46]. The ratio of HA relative to NP and M1 of the 6:2R viruses quantified by IDMS provided a complex set of results that will require further investigation to determine their significance as a parameter for selection of optimized CVV for vaccine manufacture.

The native morphology of influenza virions includes spherical, elliptical, or filamentous particles (S2 Fig), depending on the virus isolate and the host system [26]. The impact of virion morphology on the stability of particles during manufacturing is not well understood but vaccine manufacturers prefer candidate vaccine viruses that yield spherical particles [47]. Unstable filamentous virions are produced as a result of mutations in the M2 cytoplasmic tail [48]. Our electron microscopy studies evaluated virions fixed in the original allantoic fluid harvest to avoid ultracentrifugation artifacts [49] but established a stringent cutoff to account for additional deformation resulting from mechanical forces acting during virus concentration and purification. Morphometric analysis of five 6:2R viruses revealed a broad range (42 to 91%) in the proportion of spherical virions, but morphology was not associated with the HA-NA subtype of the viruses. In contrast, virion morphology was correlated with the origin of PR8 internal genes (P<0.01). M1, M2 and NP proteins have been shown to affect the spherical/filamentous morphology [27,43,50,51,52]. Although the 3 lineages of PR8 M genes encode M1 proteins with identical amino acid sequences, their M2 proteins are different at two positions, whereas their NPs vary at four positions. Further studies are needed to elucidate their possible contributions to variation in virion morphology and stability [48]. A recent report suggested a fitness advantage for growth of spherical influenza viruses in embryonated eggs [50] although spherical virions were not required for high growth in eggs [50]. In our study, non-spherical virions appeared to be correlated with the best yield from the HAY H3N2v and H3N2 6:2R viruses analyzed. In contrast, the highest yields from LAY 6:2R viruses were observed among highly spherical PR8-A viruses (e.g. Hubei/1/2010). Nevertheless, these studies indicated that a high percentage of non-spherical particles (e. g. ≥60%) is compatible with satisfactory yields of HA from the A(H3N2)v PR8-B reassortant. However, antigen losses during filtration steps used in some vaccine manufacturing processes remained to be determined since our virus purification method did not include filtration.

Sequence analysis of the PR8 lineages indicated that the internal gene proteome of PR8-A had 26 and 25 amino acid differences relative to PR8-B and PR8-C, respectively, in contrast to only five amino acid differences between the latter two donors. These differences correlated well with the HA yield results indicating distinct functional properties of PR8-A relative to the other two PR8 donor viruses. Reassortment studies followed by amino acid mutagenesis will be necessary to map the role of specific amino acid substitutions in the performance characteristics of the PR8-A, since all the genes encoded more than one amino acid polymorphism. Notably, the unique amino acid constellations in each of the three PR8 PB1 protein lineages should be dissected as this gene was reported to influence HA yield [14,28,29,53]. NP polymorphism could also be significant due to its demonstrated role in virion assembly [43]. In contrast, genetic analysis of performance differences between reassortant viruses with PR8-B and PR8-C internal genes is less complex since the products of the PB2 and the M genes are identical. These studies may also reveal the need to evaluate the potential role of non-coding nucleotide polymorphisms, which were not analyzed in this study.

Our findings complement recent work to restore the antigen yield of A/Vietnam/1194/2004 (H5N1) 6:2R viruses by repairing an assembly defect imparting low HA content in virions [54]. These studies showed that replacement of the transmembrane/cytoplasmic tail and non-coding region of HA with that of PR8 substantially improved the HA content of virions [44,54]. HA chimerization also improved the antigen yield of the A/California/7/2009 (H1N1)pdm09 NIBRG-121 virus [11,44] by enhancing growth in eggs rather than virion HA content; therefore, further studies are needed to determine the broad applicability of this approach. Another study showed that the antigen yield of a 6:2R virus derived from A/Texas/05/2009 (H1N1)pdm09 viruses was increased by replacing the transmembrane domain of NA with that of PR8 which reduced incorporation of NA and increased HA content of virions [13].

The remarkably high frequency of low antigen yield (LAY) viruses identified in the course of this study illustrates one of the major challenges facing pandemic vaccine manufacture. This study indicates that this risk could be mitigated in part by reassortment with PR8 backbones from different lineages to maximize yields from candidate vaccine viruses for egg manufacturing platforms. However, achieving satisfactory HA yields in manufacturing may require screening several wiltype HA/NA surface gene donor viruses and capitalizing on adaptive changes; which are generally selected by serial egg passage of the CVV with the most productive PR8 genes[16]. It is also worth noting that some licensed influenza vaccines that are manufactured in cultured cells; e.g. MDCK, also use the PR8-derived CVVs utilized for egg-based vaccines [55]. Studies comparing growth of 6:2R viruses to parental wild-type viruses are needed to determine whether PR8 genes could also enhance HA yield in cell culture vaccine manufacture.

In summary, this study identified important differences between three different PR8 virus lineages with respect to the antigen yields of 6:2R genotype viruses in eggs. The PR8 lineages imparting the optimal yields from 6:2R showed an association with the origin of the surface genes and their initial HA yield. Since the subtype and lineage of the HA-NA surface genes were major determinants of antigen yield, the parental donor virus should be carefully evaluated.

These findings also provide a rationale for expediting development of high-yield CVV by simultaneously generating 6:2 reassortants with three PR8 backbones, followed by IDMS analysis to identify the highest HA producer. Early selection of a high-yield reassortant as CVV will likely avert the need for further optimization and speed up pandemic vaccine manufacturing.

## Supporting Information

S1 Fig Hemagglutination (A) and egg infectious titers (B) of reassortant viruses propagated in embryonated eggs. (PDF)

S2 Fig Electron micrograph of negatively stained spherical and nonspherical virions from allantoic fluid. Scale bar corresponds to 100 nm. Asterisks denote non-spherical virions.
(PDF)

S1 Table Genetic mutations introduced to the HA gene of reverse genetics plasmids. (PDF)
