# Supplementary material for: Identification of Influenza A/PR/8/34 Donor Viruses Imparting High Hemagglutinin Yields to Candidate Vaccine Viruses in Eggs
Source: PLoS One. 2015 Jun 11;10(6):e0128982. doi: 10.1371/journal.pone.0128982 (PMC4465931; doi:10.1371/journal.pone.0128982)
Supplement: S2 Fig — Scale bar corresponds to 100 nm. Asterisks denote non-spherical virions. (PDF) [file pone.0128982.s002.pdf]

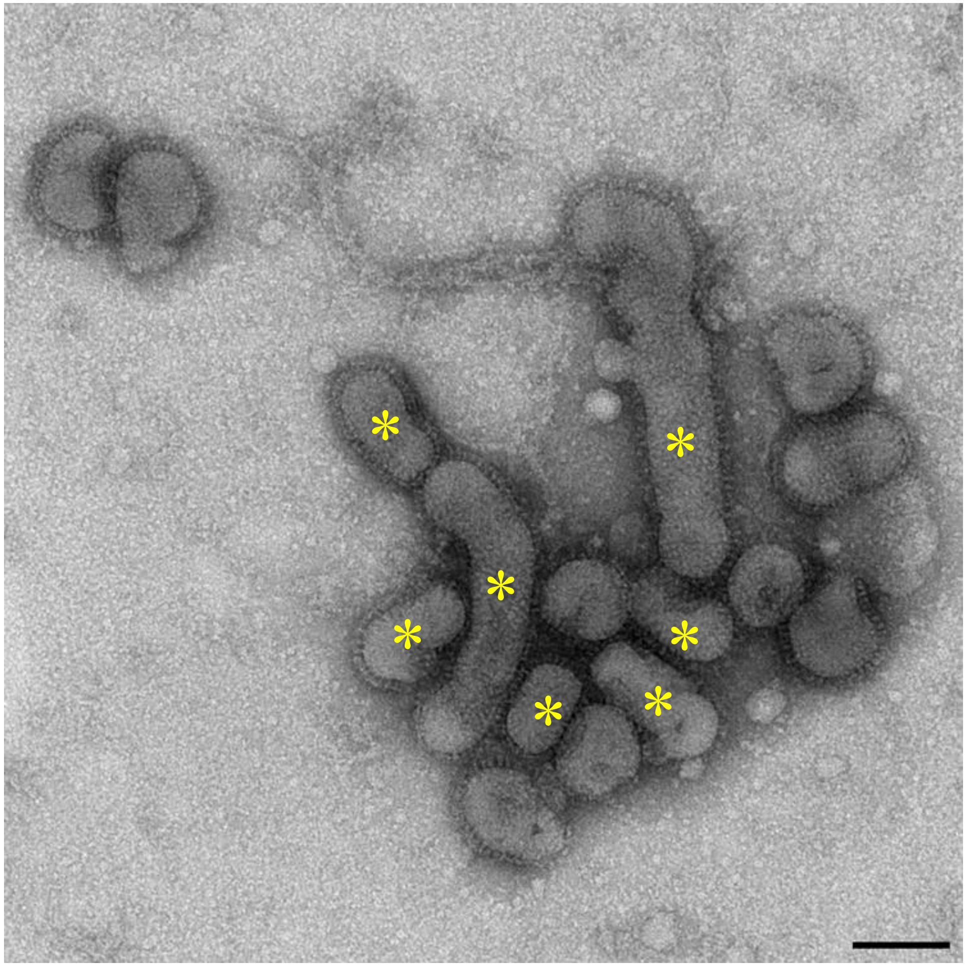

S2 Figure. Electron micrograph of negatively stained spherical and nonspherical virions from allantoic fluid.
