# Supplementary material for: Identification of Influenza A/PR/8/34 Donor Viruses Imparting High Hemagglutinin Yields to Candidate Vaccine Viruses in Eggs
Source: PLoS One. 2015 Jun 11;10(6):e0128982. doi: 10.1371/journal.pone.0128982 (PMC4465931; doi:10.1371/journal.pone.0128982)
Supplement: S1 Table — (PDF) [file pone.0128982.s003.pdf]

S1 Table. Genetic mutations introduced to the HA gene of reverse genetics plasmids

Virus Donor of surface genes	Mutation in the HA plasmid*
A/New York/18/2009	D222G
A/Hawaii/07/2009	H183L
A/Bangladesh/5071/2011	H156Q, G186V, S219Y
A/Indiana/10/2011	L194I

*amino acid numbering corresponds to mature protein
